# Pathogenesis and Potential Therapeutic Targets for Triple-Negative Breast Cancer

**DOI:** 10.3390/cancers13122978

**Published:** 2021-06-14

**Authors:** Chia-Jung Li, Yen-Dun Tony Tzeng, Yi-Han Chiu, Hung-Yu Lin, Ming-Feng Hou, Pei-Yi Chu

**Affiliations:** 1 Department of Obstetrics and Gynecology, Kaohsiung Veterans General Hospital, Kaohsiung 813, Taiwan; nigel6761@gmail.com; 2 Institute of BioPharmaceutical Sciences, National Sun Yat-sen University, Kaohsiung 804, Taiwan; 3 Department of Surgery, Kaohsiung Veterans General Hospital, Kaohsiung 813, Taiwan; 4 Institute of Biomedical Sciences, National Sun Yat-sen University, Kaohsiung 804, Taiwan; 5 Department of Microbiology, Soochow University, Taipei 111, Taiwan; chiuyiham@scu.edu.tw; 6 Research Assistant Center, Show Chwan Memorial Hospital, Changhua 500, Taiwan; linhungyu700218@gmail.com; 7 Center for Cancer Research, Division of Breast Surgery, Department of Surgery, Kaohsiung Medical University Chung-Ho Memorial Hospital, Kaohsiung 807, Taiwan; mifeho@kmu.edu.tw; 8 School of Medicine, College of Medicine, Fu Jen Catholic University, New Taipei City 242, Taiwan; 9 Department of Pathology, Show Chwan Memorial Hospital, Changhua 500, Taiwan; 10 Department of Health Food, Chung Chou University of Science and Technology, Changhua 510, Taiwan; 11 National Institute of Cancer Research, National Health Research Institutes, Tainan 704, Taiwan

**Keywords:** breast cancer, target therapy, biomarkers, pathogenesis

## Abstract

**Simple Summary:**

Breast cancer emergencies have become a rapidly evolving field in medicine during the last ten years. Carcinogenesis is a multiparametric process that involves diverse factors such as genetic, environmental, or aging. Recent research that elucidates the tumor biology and molecular pathways that mediate cancer progression and drug resistance has led to the development of various molecular targeted therapies involving monoclonal antibodies, small molecule receptor tyrosine kinase inhibitors, and agents that block downstream signaling pathways in breast cancer.

**Abstract:**

Triple negative breast cancer (TNBC) is a heterogeneous tumor characterized by early recurrence, high invasion, and poor prognosis. Currently, its treatment includes chemotherapy, which shows a suboptimal efficacy. However, with the increasing studies on TNBC subtypes and tumor molecular biology, great progress has been made in targeted therapy for TNBC. The new developments in the treatment of breast cancer include targeted therapy, which has the advantages of accurate positioning, high efficiency, and low toxicity, as compared to surgery, radiotherapy, and chemotherapy. Given its importance as cancer treatment, we review the latest research on the subtypes of TNBC and relevant targeted therapies.

## 1. Introduction

Breast cancer is the most common malignancy among women with an incidence rate of 10,492 in 100,000 cases in 2015, according to reports of the Ministry of Health and Welfare. The high morbidity and mortality rates cause a financial burden with regards to health insurance [1]. Several risk factors for breast cancer were investigated, including alcohol intake (relative risk: 1.10; 95% confidence interval: 1.06–1.14) [2], obesity (hazard ratio: 1.58, 95% confidence interval: 1.40–1.79) [3], age (hazard ratio: 10.1, 95% confidence interval: 8.49–11.94) [4], early menarche, and late menopause. Despite several years of bench to bedside investigations, breast cancer remains a challenge for all physicians, with the detailed mechanism of its tumorigenesis still unclear. Its molecular and biological attributes were investigated in detail to better understand the development and pathogenesis of breast cancer, including oncogenes, the tumor suppression genes, and downstream pathways that promote tumor proliferation, differentiation, and distant metastasis. The treatment of breast cancer requires a multidisciplinary approach involving surgery, radiation, and medical oncology to control tumor cell proliferation and metastasis. The new treatments for breast cancer are also discussed in this article, including the use of PARP, MAPK, CHK1, CDK 4/6 inhibitor, anticytotoxic T-lymphocyte-associated protein 4 (anti-CTLA4) antibodies, and anti-programmed cell death protein 1 (anti-PD1)/ligand 1 (anti-PD-L1) antibodies. We believe that elucidating the mechanism of breast cancer will provide an important foundation for therapeutic intervention. This article provides an update on research and clinical results using the molecular biology of tumorigenesis and summarizes current treatments to provide a new strategy for physicians.

## 2. Clinical Features of Breast Cancer

The common clinical presentation of breast cancer is usually a palpable breast mass observed upon on self-breast examination. Other associated symptoms included local pain or nipple discharge. In metastasis, fever, weight loss, night sweats, or bone pain may be present as a result of tumor progression. Imaging studies play an important role in early detection and timely intervention. Three image detection modalities for breast cancer were investigated, including breast ultrasound, mammography, and magnetic resonance imaging (MRI). A breast ultrasound is effective for characterizing malignant solid masses as an initial diagnostic evaluation with a sensitivity rate of 98.4% and negative predictive value of 99.5% [5,6]. The mammogram was used for elderly women to characterize soft tissue density, which may present with an irregular border, spiculated pattern, and local calcifications. According to mammographic findings, the radiologist will utilize the Breast Imaging Reporting and Data System (BI-RADS) for further diagnostic assessment (Table 1). The breast MRI is an important tool in preoperative survey and postoperative follow-up, especially to evaluate scar tissue. The sensitivity is reported to be high at more than 90%, but the specificity is moderate at about 72%. In high-risk breast cancer screening, breast MRI has an important role in preventing missed diagnoses [7]. Several prognostic and predictive factors for breast cancer were reported, including the tumor node metastasis (TNM) system, tumor morphology, and tumor markers. The TNM system categorizes breast cancer by location and invasion anatomy, including tumor size, lymph node involvement, and distant metastasis. The eighth edition of the TNM system is shown in Table 2, reported from the Union for International Cancer Control (UICC) in 2017.

The anatomic stage provides surgeons with important information needed for making decisions about surgical intervention and preventing complications. After surgical intervention, the target therapy and chemotherapy play a critical role in controlling tumor proliferation, local recurrence, and distant metastasis. The tumor biology was investigated and is important in prognosis and response to treatment. Biomarkers have been investigated and taken into account in modified staging systems to accurately predict clinical outcomes. The expression of estrogen receptor (ER), progesterone receptor (PR), and human epidermal growth factor receptor 2 (HER2) are generally used in clinical management. The expression of hormone receptors, including ER and PR, are reflective of the response to adjuvant endocrine therapy and are associated with better outcomes. Currently, the expression of ER and PR levels is significantly related to the overall survival, disease-free survival, and time to treatment failure [8]. The status of ER expression is associated with the distant metastatic site. Bone and soft tissue are common distant metastatic sites of ER-positive breast cancer, compared to ER-negative breast cancer, which usually occurs in the brain and liver. The expression of the PR is independent of ER expression and also significantly related to clinical outcome. PR-negative breast cancers have a more aggressive prognosis. HER2 overexpression is a predictor of poor clinical outcomes if patients do not receive chemotherapy and target therapy. Other biomarkers, including Ki-67, luminal A, luminal B, and basal, are involved in clinical prognosis. In high-grade breast cancer with rapid progression, the mitotic count and expression of Ki-67 are elevated, reflecting a high proliferation rate, and this type of cancer may better respond to anthracycline treatment, which inhibits DNA and RNA synthesis. The expression of luminal A and luminal B genes is associated with ER-positive breast cancers. The luminal subtype tumors typically express luminal cytokeratin 8 and 18. Luminal A tumors are usually associated with high expression of ER and lower expression of HER-2 genes. Luminal B tumors are associated with lower expression of the ER gene and have poor prognosis, compared to the luminal A subtype. With the emergence of genomics and transcriptomics techniques, several thousands of genes have been identified and investigated to predict clinical prognosis. The detailed mechanisms of tumorigenesis and the interaction with the tumor microenvironment are discussed below.

## 3. Molecular Types of Triple Negative Breast Cancer

**Figure 1:**
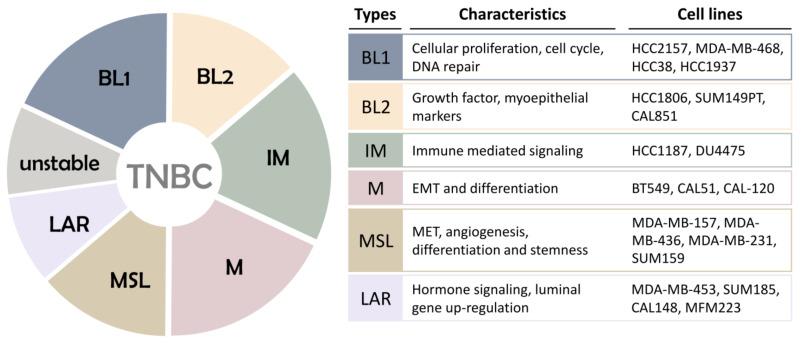
Association of different TNBC subtypes. TNBC gene expression subtypes are related to unique molecular characteristics and different TNBCs. Subtypes of TNBC: BL1, basal-like 1; BL2, basal-like 2; IM, immunomodulatory; ML, mesenchymal-like; MSL, mesenchymal stem-like; LAR, luminal androgen receptor.

Breast tumor heterogeneity is one of the main reasons for the significant differences in the therapeutic effects of breast cancer patients. In 2011, Lehmann’s team examined the genetic sequence of TNBC patients through mRNA expression profiling clustering and found that TNBC can be classified into six subtypes: basal cell-like type 1 (BL1), basal cell Like type 2 (BL2), immunomodulatory subtype (IM), mesenchymal subtype (M), mesenchymal stem cell-like subtype (MSL), and luminal androgen receptor subtype (LAR) [9]. These six subtypes have different gene expression profiles and are involved in different signal transduction pathways (Figure 1). The BL1 and BL2 subtypes account for about 50% of TNBC. The BL1 subtype has high expression of cell proliferation-related genes, DNA damage repair genes, and Ki-67 protein. The BL2 subtype has high expression of multiple growth factor signal pathway genes and metabolic signal transduction genes. IM subtypes have high expression of multiple immune-related signal pathways and cytokine-related signal pathways. M and MSL subtypes have high expression of epithelial–mesenchymal transition (EMT) genes and cell differentiation-related genes, and are sensitive to PI3K/mTOR inhibitors [10]. The difference between M-type and MSL-type TNBC is the low expression of cell proliferation protein and tight junction protein in the MSL subtype, and the low mitotic index. The M subtype is related to cell motility, cell differentiation, and extracellular matrix receptors. LAR subtypes are rich in hormone-regulated signaling pathways, including steroid synthesis and androgen receptor signaling pathways.

Further classification of TNBC can bring more accurate diagnosis and personalized treatment to TNBC patients. In 2015, Burstein et al. performed a matrix analysis of gene expression in 198 TNBC patient microarrays at Baylor College of Medicine, USA, and grouped 80 core genes to obtain four more stable TNBC subtypes, namely basal like/immune suppressed (BLIS), basal like/immune activated (BLIA), mesenchymal (MES), and LAR [11]. Liu et al. in their 2016 study stated that by typing analysis of 145 TNBC cases, the superior typing method was obtained by classifying TNBC into four subtypes: BLIS, IM, MES, and LAR [12]. Lehmann’s team again refined the TNBC molecular subtypes from six (TNBCtype) to four (TNBCtype-4) tumor-specific subtypes (BL1, BL2, M, and LAR) in 2016 and demonstrated differences in diagnostic age, grading, local and distant disease progression, and histopathology [13]. In addition, in 2019, Kim et al. first classified TNBC into neutrophil-enriched subtype (NES) and macrophage-enriched subtype (MES), and found that immune checkpoint blockage (ICB) therapy has different efficacy on these two subtypes [14], providing a new idea for molecular typing of TNBC to guide clinical treatment. The subtypes and classification of different TNBC tumors should provide important value for future clinical decision making and for TNBC patients with conventional chemotherapy versus targeted and immunotherapy therapies currently in clinical trials.

Although the TNBC molecular classification defines four major subtypes, BL1 and BL2, M, and LAR, with distinct ontologies and different responses to therapy, the LAR definition seems to be one of the most reliable definitions among the different gene ontology-based classification systems. There are different classification systems based on DNA and RNA analysis that identify four molecularly defined TNBC subtypes: LAR, mesenchymal (MES), basal-like immunosuppression (BLIS), and basal-like immune activation (BLIA), characterized by different prognosis and potential therapeutic targets [11,15]. Interestingly, DNA analysis revealed specific gene amplification and targetable molecular expression. LAR was also found to be characterized by AR and MUC1 markers, suggesting the strategic importance of anti-AR therapies and the potential role of MUC1 vaccines as an effective treatment for this subtype [16]. Compared to other profiles, the LAR subtypes identified by this new classification system share the same genetic and biological features as those identified by Lehmann [9] and Pietenpol et al. [9,11].

## 4. Prognostic Biomarkers of TNBC

### 4.1. BRCA 1/2

*BRCA1* and *BRCA2* are human tumor suppressor genes located on the long arm of chromosomes 17 and 13, respectively. The structures of *BRCA1* and *BRCA2* are different, but their roles are related and important in the DNA repair process [17]. *BRCA2* binds to a DNA single strand and directly affects the regulation of recombinase, whereas *RAD51* localizes to DNA double-strand cleavage, which requires the formation of the *BRCA1–PALB2–BRCA2* complex. Therefore, *BRCA1/BRCA2* encodes a protein that maintains human genome stability and prevents mutant genes. The rearrangement is significant [18,19]. *BRCA1/BRCA2* plays an important role in DNA double-strand break repair. *BRCA1/BRCA2* is involved in the repair of damaged DNA. If the repair of DNA is inhibited, it will induce apoptosis to clear the cells that may be mutated; however, once *BRCA1/BRCA2* is mutated, DNA repair cannot be completed, and the progression of tumor development is promoted [20]. Mutations in *BRCA1/BRCA2* are recognized as risk factors for breast cancer induction, but the role in breast cancer prognosis is still controversial. Existing studies seem to favor *BRCA1/BRCA2* as having little effect on the prognosis of breast cancer. Previous studies have indicated that there is no difference in overall survival between BRCA-associated breast cancer and sporadic breast cancer [21]. Similarly, there is a meta-analysis showing that the evidence from 66 studies does not support the poor prognosis of *BRCA1/BRCA2* mutation carriers. The study analyzed the relationship between *BRCA1* and *BRCA2* single mutation carrying and double mutation carrying with recurrence-free survival, metastasis-free survival, and overall survival in breast cancer patients, which were not associated with clinical factors after adjustment [22]. Therefore, the role of BRCA mutations in breast cancer progression and BRCA mutation-targeted treatment effects remain to be confirmed by large-scale, multicenter, prospective studies.

### 4.2. ALDH1A1

The protein encoded by *ALDH1A1* (aldehyde dehydrogenase 1 family member A1) belongs to the aldehyde dehydrogenase family [18]. Aldehyde dehydrogenase is a polymorphic enzyme responsible for the oxidation of aldehydes to carboxylic acids, which leave the liver and are metabolized by the body’s muscle and heart [23]. ALDH1A1 is also an important component of corneal lens protein, which helps maintain the transparency of the cornea. In the face of cancer, ALDH1A1 is regarded as a marker of breast cancer stem cells, and cancer stem cells have strong carcinogenicity and self-renewal ability, and play an important role in tumorigenesis, development, and prognosis [24]. Tan et al. showed that *ALDH1A1* is highly expressed in breast cancer tissues, but it is not associated with prognosis. *ALDH1* is expressed in 25% of tumors, and the *ALDH1*-positive rate in young patients is almost 14 times higher than that in elderly patients. *ALDH1A1* is considered to be an independent ER-negative factor, but it is not associated with breast cancer recurrence and associated death [25]. Numerous studies suggest that the expression of *ALDH1A1* suggests a poor prognosis for breast cancer [24,26]. Japanese studies have found that 26% of patients with invasive ductal carcinoma have *ALDH1* expression, which is associated with larger tumors, higher histological grades, later TNM stage, HER2 overexpression, and hormone receptor negative status. The recurrence-free survival time and overall survival time of the *ALDH1A1* expression group were shorter than those of the non-expression group [27]. The results of a meta-analysis of a total of 3274 breast cancer patients enrolled in 15 studies were consistent with those of the Japanese study. In addition, cases with *ALDH1A1* expression are more likely to show development of lymph node metastasis, confirming that *ALDH1A1* is a marker of poor tumor progression and prognosis [24].

A previous study pointed out that Ma et al. [28] showed shorter recurrence-free survival (RFS) and overall survival (OS) in survival analysis of *ALDH1*-expressing patients in 158 cases of TNBC. Ying et al. [24] reported that *ALDH1* expression is a biomarker for predicting poor survival in breast cancer patients. However, Morimoto et al. [29] found no statistical difference in disease-free survival (DFS) and OS between *ALDH1^+^* and *ALDH1^-^* cases in TNBC patients. Meanwhile, Resetkova et al. [30] showed that stromal expression of ALDH1 rather than tumor expression was associated with TNBC survival. Although there are differences and controversies between these studies, this may be due to different case characteristics, and assay sensitivity and assessment criteria, and sample size. The small number of relevant studies may also explain the lack of consistency in results. Regardless of the reasons for the discrepancies, further studies involving larger cohorts and using standardized and well-matched controls are needed to validate these results.

### 4.3. CXCR4

*CXCR4* (C-X-C chemokine receptor type 4) is expressed in most tissues and parenchymal organs in the body and is a G protein-coupled receptor (GPCR) consisting of 352 amino acids. The chemokine receptor *CXCR4* is a specific receptor for chemokine (C-X-C) ligand 12 (CXCL12, also known as chemokine stromal cell-derived factor-1) [31]. CXCL12 has a strong chemotactic effect on lymphocytes, and *CXCR4* plays an important role in various physiological functions, such as the dynamic balance of immune cells, including T cells. In tissue regeneration, the CXCL12/CXCR4 system plays an important role in promoting stem cell homing [32].

*CXCR4* has a clear suggestive effect on breast cancer metastasis and prognosis, and is highly expressed in a variety of tumor cells and involved in chemotaxis, invasion, angiogenesis, and cell proliferation. It has been shown that breast cancer patients with high *CXCR4* expression have poor prognosis [33,34]. In breast cancer patients, *CXCR4* overexpression is associated with lymph node status and poor prognosis [33]. In addition, almost 75% of TNBC patients exhibit high *CXCR4* expression [34]. Furthermore, there is a strong association between *CXCR4* overexpression and histological cancer grade in menopausal and TNBC patients. The five-year DFS and five-year OS rates were 57.7% and 58.3% for the low *CXCR4* group and 42.1% and 44.7% for the high *CXCR4* group, respectively [35]. Patients with high *CXCR4* overexpression (≥6-fold) in TNBC had a significantly higher incidence of cancer recurrence and cancer-related death than the low *CXCR4* group (<6-fold) [34]. This result indicates that overexpression of *CXCR4* in cancer specimens predicts a poor prognosis for TNBC and may be a predictor of poor prognosis.

A meta-analysis of the results of 15 studies (3104 patients in total) found that the expression of *CXCR4* in breast cancer tissues was significantly higher than that in adjacent tissues, and high expression of *CXCR4* in whole cells or the cytoplasm often indicated poor prognosis of breast cancer [36]. Another systematic review confirms these findings and shows that *CXCR4* overexpression is significantly associated with lymph node metastasis and distant metastasis, with lower overall survival and disease-free survival. There is no correlation with clinical features of breast cancer, tumor stage, ER, and PR [32]. However, because of the inevitable omission of the meta-analysis literature search process and the differences among different research populations, understanding the specific mechanism of action of *CXCR4* and whether it can be changed by radiotherapy and chemotherapy still needs further research.

### 4.4. p16^INK4a^

Inactivation of multiple tumor suppressor genes plays a role in the transformation of normal breast tissue into cancer. According to the multiangle analysis of tumor pathological type, grade of malignancy, efficacy, and prognosis, p16^INK4a^ also plays an important role. There is a high deletion rate of *p16^INK4a^* in breast cancer cell lines. The main inactivation modes are deletion, point mutation, abnormal expression, and CpG island methylation in the promoter region [37,38]. Tumor suppressor genes often exhibit high frequency deletions and mutations in tumor cells. It was previously thought that abnormal changes in the *p16^INK4a^* gene were dominated by gene deletion, and point mutations were not the main cause of genetic changes. The overexpression rate of *p16^INK4a^* is more prominent in tumors with higher malignancy, and it is more overexpressed in breast cancer [39]. Conversely, there have been reports of *p16^INK4a^* downregulation in breast cancer [40].

The *p16^INK4a^* gene often has low expression in normal human tissue cells, and p16^INK4a^ protein is generally not detected. The abnormal expression of *p16^INK4a^* may be related to the occurrence and development of breast cancer. However, whether it can become an independent prognostic factor or a high specificity marker for breast cancer remains to be confirmed by further large-scale clinical analysis and prospective studies. Recent studies have indicated that the methylation of CpG islands in the *p16^INK4a^* promoter region is more frequent than that of point mutations and homozygous deletions, and may be more valuable for the mechanism of p16 methylation induction and reversal. The methylation rate of the *p16^INK4a^* in breast cancer was found to be 30%, which is consistent with the results of two different research teams. It was also suggested that the methylation rate of breast cancer tissue was significantly higher than that of normal tissues and correlated with lymph node metastasis. The difference was statistically significant, but it was not significantly associated with the degree of differentiation of breast cancer [41,42]. These studies have shown that the higher methylation rate of the *p16^INK4a^* has important significance for breast cancer. The detection of the methylation rate of the *p16^INK4a^* may have high clinical value for early diagnosis of breast cancer. Moreover, the inhibition of tumor growth by the *p16^INK4a^* may play a role in anti-angiogenesis and induction of tumor cell apoptosis, in addition to mediating the arrest function of the cell proliferation cycle [43].

In addition, high *p16^INK4a^* expression has been shown to be associated with aggressive behavior and poor outcome in TNBC in studies of TNBC; thus, p16^INK4a^ has potential prognostic utility and predictive value. High expression of *p16^INK4a^* predicts progression from in situ ductal carcinoma to basal-like invasive breast cancer [44]. It was also reported that *p16^INK4a^* overexpression was positively correlated with *Ki67* expression in *p53*-negative TNBC but not in p53-positive tumors, suggesting that patients with p53-negative TNBC with high *p16^INK4a^* expression have a poorer prognosis [45]. Moreover, diffuse *p16^INK4a^* immunostaining in most cases of basal-like breast cancer and TNBC and a positive correlation between Rb negativity and diffuse *p16^INK4a^* expression was found [44]. Previous studies have found a better prognostic correlation for low *p16^INK4a^* expression in tumor tissues of TNBC cancer patients [46]. However, there was no prognostic significance between *p16^INK4a^* and overall survival and recurrence-free survival, in 60 TNBC patients [47]. As evidenced by these findings, there are conflicting data regarding the correlation between *p16^INK4a^* and prognosis, and more studies are needed to fully define the role of *p16^INK4a^* in TNBC clinical outcomes and survival.

### 4.5. ATM

*ATM* is a gene that is mutated in ataxia telangiectasia (A-T) and has been associated with cancer since its discovery. Swift et al. found that patients heterozygous for *ATM* genes are more likely to develop breast cancer; *ATM* genes are being explored widely for their role in gene function [48]. Although many studies in homozygous patients have failed to find links between *ATM* genes and tumors, this association has been confirmed in *ATM* heterozygotes, particularly the link between *ATM* heterozygotes and breast cancer. Western women have a 10% to 12% risk of breast cancer in their lifetime. The family history of breast cancer plays a key role in the pathogenesis. In addition, 5–10% of breast cancer patients have high penetrance germline gene mutations. The most important of these are the breast cancer susceptibility genes *BRCA1* and *BRCA2*, and *ATM* and *CHK2* are also important risk factors [49]. The role of the *ATM* gene in familial breast cancer is based on the exclusion of the susceptibility genes *BRCA1*, *BRCA2*, and *CHK2*. The detection rate of mutations in the *ATM* gene was 12/443 in the case group and 2/521 in the control group. This result suggests that mutations in the *ATM* gene may increase the incidence of breast cancer [50]. In addition to studies of *ATM* polymorphisms and gene mutations, recent studies have suggested that *ATM* mRNA levels are associated with lower metastasis-free survival (MFS), and low-level expression of ATM protein results in a shorter MFS. miR-203 is a dominant indicator of downregulation of the *ATM* gene in breast cancer. *ATM* gene mRNA and ATM protein levels are independent prognostic factors for sporadic breast cancer and play a good guiding role in treatment [51].

The *ATM* gene encodes a phosphatidylinositol 3-kinase (*PI3K*) that regulates and participates in the repair of DNA damage and a key substrate for cell cycle control. Previous studies analyzing the *ATM* gene in European women (42,671 cases and 42,164 controls) found an association between *ATM* mutations (c.7271 T > G) and overall breast cancer risk; however, a specific association with TNBC could not be determined because the tumors were not stratified by subtype [52]. In addition, 158 women with TNBC were recruited for analysis among Polish women, one of whom carried an *ATM* mutation, while no *ATM* mutation was detected in 44 women with non-TNBC hereditary breast cancer [53]. Other studies have observed an enrichment of *ATM* mutations in patients with ER-positive tumors [54,55], with a five-fold increase in *ATM* mutations in non-TNBC patients compared to TNBC tumors [56]. At present, the discussion regarding the ATM gene is of great value in the diagnosis, treatment, and prognosis of breast cancer. Determining whether it is a biomarker of breast cancer susceptibility like *BRCA1* and *BACA2* is worth investigating.

### 4.6. PTEN

The *PTEN* gene is an important tumor suppressor gene and plays a key regulatory role in breast cancer. In animals, it was found that 49% of female mice developed breast cancer six months after *PTEN* gene knockout (*PTEN^+/−^*) [57]. The effect of stable transfection of exogenous *PTEN* on the proliferation of endogenous PTEN-deficient human breast cancer cells confirmed that *PTEN* significantly inhibited the proliferation of breast cancer cells [58]. Therefore, determining whether the *PTEN* gene can inhibit the proliferation of tumor cells when artificially introduced into the cells of breast cancer patients could provide a new avenue for treatment. Through high-resolution fluorescence microsatellite analysis, it was found that *AKT* activation was positively correlated with *PTEN* gene deletion and *HER-2* overexpression in breast cancer patients and negatively correlated with PR. Simultaneous deletion of *PTEN* and *HER-2* overexpression results in enhanced activation of *AKT*, leading to the possibility of PR-negative expression [59]. The total positive expression rate of the *PTEN* gene in breast cancer was 69%, and the expression level in invasive breast cancer was significantly lower than that in breast carcinoma in situ and benign proliferative lesions. The positive expression rate of *PTEN* gradually decreases as the tumor progresses [60]. Breast cancer with downregulated PTEN protein has a strong invasiveness and is prone to infiltration and metastasis [61]. The expression of the *PTEN* gene can be used as a potential prognostic indicator.

*PTEN* mutations are common in TNBC, with gene-mediated loss of function occurring in 15% of cases [62] *PTEN* has recently been shown to protect the genome from instability by maintaining chromosomal integrity. Although women with Cowden syndrome carrying *PTEN* germline mutations have a 50% lifetime risk of developing breast cancer [63], there is no consistent phenotype of breast cancer associated with *PTEN* mutations. Most PTEN-associated tumors are more likely to be tubulointerstitial than TNBC. Observations suggest that (1) there is no significant difference in the incidence of *PTEN*-causing mutations between women with TNBC (*n* = 692) and women with non-TNBC tumors (*n* = 2696) [56]; and (2) the observation of only one deleterious mutation in 267 women [64] supports the view that *PTEN* mutations are not associated with an increased risk of TNBC. Preclinical AKT inhibition has higher activity in PTEN-deficient models of cancer [65], but there are significant challenges in determining which *PTEN* mutations confer sufficient loss of function to be targetable and the best way to identify such loss of function.

## 5. Therapeutic Strategy for TNBC

### 5.1. HER2 Inhibitor

Since the first discovery of *HER-2* in breast cancer, its importance in the occurrence and development of breast cancer has gradually been recognized. Current targeted therapy research focuses on blocking the *HER-2* signaling pathway. Trastuzumab, a recombinant DNA-derived humanized monoclonal antibody, is a novel drug for the treatment of metastatic breast cancer. Trastuzumab has achieved good results in both *HER-2*-positive early and late-stage breast cancer treatments. Trastuzumab is used alone or in combination with chemotherapy for patients with advanced breast cancer. Trastuzumab treatment can prolong patient survival with low side effects. In a further in-depth study, the results of a combination chemotherapy regimen using trastuzumab with docetaxel or taxane resulted in overall survival of patients that was higher than that obtained using chemotherapy alone [66]. Therefore, trastuzumab combined with paclitaxel is the first-line treatment for *HER-2* positive breast cancer patients. The investigators conducted a four-year follow-up study showing that patients who received trastuzumab in the observation group were more likely to have a higher survival rate (including disease-free survival) than those who did not receive treatment [67]. Since the patient’s treatment depends on his or her personal will, it is impossible to carry out rigorous statistical analysis, but patients receiving treatment have increases in disease-free survival and overall survival.

*HER-2*-targeted therapy has not benefited patients with low *HER-2* expression in TNBC treatment strategies; however, combination therapy may be efficacious. The primary analysis of a phase IIb trial investigating the HER2-derived vaccine nelipepimut-S (NPS) did not benefit the intention-to-treat population (NPS; *n* = 55 vs. placebo; *n* = 44), but a subgroup analysis showed benefit in patients with TNBC [68]. Another study enrolled 136 people receiving NPS/GM-CSF and 139 people receiving placebo/GM-CSF. The results of the study confirmed that concomitant administration of trastuzumab and NPS with GM-CSF was safe and had no additional overall toxicity. The combination of *HER-2*-targeted NPS and trastuzumab was safe. In *HER-2* low-expressing breast cancer, no significant differences in DFS were observed in the intention-to-treat analysis [69]; however, significant clinical benefits were observed in TNBC patients. These findings warrant further investigation in a phase III randomized trial.

#### Targeting RTK Signaling

Lapatinib is an RTKI that reversibly blocks *EGFR* and *HER-2* [70], inhibits downstream *MAPK/Erk1/2* and *PI3K/AKT* pathways [71], and enhances trastuzumab-dependent cell-mediated cytotoxicity [72]. In March 2007, the FDA approved lapatinib in combination with capecitabine for *HER-2*-positive breast cancer previously treated with anthracyclines, paclitaxel, and trastuzumab [73] In February 2010, the FDA approved lapatinib in combination with letrozole for the first-line treatment of hormone receptor (HR)-positive and *HER-2* overexpressing postmenopausal metastatic breast cancer [74].

Lenatinib is a TKI that irreversibly inhibits *HER-1*, *HER-2*, and *HER-4*. Early clinical studies have shown that lenatinib exerts more effective inhibition than lapatinib in possible resistance pathways in *HER-2*-positive patients previously treated with trastuzumab or anti-HER-2. In July 2017, the FDA approved lenatinib for extended postoperative treatment with trastuzumab adjuvant therapy in patients with early-stage *HER-2*-positive breast cancer [75].

Tucatinib is a new oral selective TKI that is more selective for *HER-2* than *EGFR* [76]. Recently, the FDA announced the granting of a New Drug Priority Approval Application (NDA) for tucatinib in combination with trastuzumab and capecitabine for the treatment of locally advanced unresectable or metastatic *HER-2*-positive breast cancer, including patients with brain metastases. 

Pazopanib is a novel multitarget TKI that targets *VEGFR-1*, *VEGFR-2*, *VEGFR-3*, *PDGFR/β*, and *c-Kit*, and has been shown to inhibit tumor growth and angiogenesis in in vitro studies [77].

### 5.2. VEGF Inhibitor

TNBC is a highly proliferating tumor that requires the involvement of angiogenesis in its development, invasion, and metastasis processes. The expression level of vascular endothelial growth factor (*VEGF)* is an independent prognostic factor in early breast cancer. High expression of *VEGF* suggests high tumor malignancy, easy recurrence and metastasis, short disease-free survival, and low overall survival. *VEGF-A* is highly expressed in TNBC cells, so TNBC is generally considered to be sensitive to VEGF-targeting drugs [78]. Platelet-derived growth factor (PDGF) is one of the angiogenic factors. Abnormal activation of *PDG*F or *PDGFR* induces tumor angiogenesis and promotes migration and invasion of tumor cells. Overexpression of *VEGF* in tumor tissues can promote the migration and proliferation of vascular endothelial cells, promote angiogenesis, increase tumor growth, and significantly increase vascular permeability, allowing tumor cells to enter the blood vessels for tumor invasion and metastasis, providing more favorable conditions [79]. Currently, antiangiogenic drugs include bevacizumab and ramucirumab, and tyrosine kinase inhibitors such as sunitinib and sorafenib. These antiangiogenic drugs are likely to have higher antitumor activity in TNBC. In a phase II study of paclitaxel, bevacizumab, and gemcitabine in the treatment of *HER2*-negative breast cancer, the clinical benefit rate in the TNBC subgroup was 84.6%, and approximately 82.5% of patients achieved a total survival of 18 months [80]. In another phase III trial, RIBBON-2 in the TNBC subgroup, the bevacizumab combination group, and the chemotherapy group showed a reduction in the risk of disease progression by 51%. It also increased progression-free survival by 3.3 months and overall survival by 5.3 months [81]. These results demonstrate the efficacy of antiangiogenic drugs in inhibiting tumors in TNBC.

### 5.3. PARP Inhibitor

PARP is a key enzyme for repairing broken DNA single strands, and in wild-type cells containing *BRCA1/2*, broken DNA double strands can be repaired by homologous recombination. However, in BRCA1/2-deficient cells, the homologous recombination function fails and therefore PARP is relied upon to repair the broken DNA single strand. Therefore, PARP inhibitors have the ability to prevent self-repair in *BRCA1/2* mutated breast cancer cells and accelerate apoptosis of tumor cells, thereby enhancing the efficacy of chemotherapy as well as radiotherapy [82].

As a novel drug, PARP inhibitors can cause double-strand breaks in BRCA-mutant breast cancer cells, leading to cell death due to synthetic lethality [83]. Olaparib is an effective PARP-1 and PARP-2 inhibitor [84] and was the first to be used by the FDA and the European Medicines Agency (EMEA) (Table 3). A PARP inhibitor was clinically approved for treatment of relapsed high-grade serous ovarian cancer. Olapani was first used in the clinical study of BRCA-related breast cancer, ClinicalTrials.gov Identifier: NCT00494234. Patients received olaparib (100 mg/time, 2 times/d (*n* = 27) and 400 mg/time, twice/d (*n* = 27)), and the results indicated a dose-dependent objective response rate (ORR) of 22% (100 mg group) and 41% (400 mg group). The 400 mg group had a median progression-free survival of 5.7 months (95% CI: 4.6–7.4), and that of the 100 mg group was 3.8 months (95% CI: 1.9–5.5) [85]. After a preliminary determination of the therapeutically effective dose, the OlympiAD study became the latest basis for FDA-approved olrapani, which included *BRCA*-mutant HER2-negative advanced breast cancer patients. The study group (*n* = 205) received olaparib (300 mg/second, 2 times/d monotherapy) while the control group (*n* = 97) only received capecitabine, eribulin, or vinorelbine in three cycle regimen single-agent chemotherapy. The study showed that the study group and the control group were 7.0 and 4.2 months (HR = 0.58, 95% CI: 0.33 to 0.80, *p* < 0.001). Therefore, the study indicated that olaparib has significant advantages in terms of efficacy and safety compared to other chemotherapeutic drugs [86].

Intractable TNBC requires more effective treatment strategies, so new drugs are being developed to sensitize sporadic TNBC to PARP inhibition. A clinical trial (ClinicalTrials.gov Identifier: NCT01623349) is evaluating olaparib and BKM120 (buparlisib) or BYL719 (PI3 kinase inhibitor) in advanced sporadic TNBC and high-grade plasma ovarian cancer [87]. Another clinical trial (ClinicalTrials.gov Identifier: NCT01434316) combined veliparib and dinaciclib (CDK inhibitor), in which CDK inhibition was assumed to sensitize TNBC to PARP inhibition [88]. After the phase I dose ascertainment portion, the study will enroll patients with *BRCA*-mutated and nonmutated advanced breast cancer in a dose expansion cohort. Another PARP inhibitor, talazoparib (BMN 673), effectively modulates *PARP* transcription. In addition, veriparib (veliparib), a novel potent PARP-1 and PARP-2 inhibitor, had a 51% partial remission rate in the treatment group in a clinical phase II trial of breast cancer treatment, which was significantly higher than the standard chemotherapy group (26%) [89]. This study suggests that veriparib is more effective than standard therapy for TNBC with a high risk of recurrence. In addition, multicenter clinical trials are evaluating the efficacy and safety of PARP inhibitors in combination with chemotherapy, immunosuppressants, and inhibitors of other targets (e.g., *VEGFR*, *HSP90*, *P13K/AKT/mTOR*, etc.) for the treatment of TNBC [90]. Regimens such as olaparib in combination with VEGFR or P13K inhibitors can effectively inhibit the rapid proliferation and growth of TNBC cells by affecting the blood oxygen supply to tumor cells and blocking molecules required for cell growth [91].

### 5.4. CHK1 Inhibitor

Checkpoint kinase 1 (*CHK1*) belongs to the serine/threonine protein kinase family and plays an important role in the regulation of cell cycle arrest by checkpoints caused by DNA damage or the presence of un-replicated DNA. The CHK1 inhibitor UNC01 abolished the production of G2 checkpoints dependent on DNA damage in cisplatin treatment and increased the sensitivity of cisplatin by nearly 60-fold. In addition, the TCGA database analysis found that the p53 mutation rate was very high in TNBC. The CHK1 inhibitor (UCN01 or AZD7762) increases the sensitivity of TNBC xenografts with *p53* mutations to chemotherapy and achieves good preclinical effects [92]. These all indicate that *CHK1* is a very promising target in patients with p53 mutations in TNBC. Based on the recognition that CHK1 is a drug target, other CHK1 inhibitors, AZD7762, PF-477736, SCH900776, and LY2606368, are undergoing phase I and II clinical trials.

### 5.5. EGFR Inhibitor

*EGFR* is a transmembrane tyrosine kinase growth factor receptor involved in cell proliferation and differentiation in normal tissues. It also participates in the adhesion and movement of normal tissue cells and initiates many downstream cell signal transduction pathways [110,111]. *EGFR* is a member of the *ErbB* family of membrane tyrosine kinase receptors. Studies have shown that most TNBCs have overexpressed *EGFR,* and early TNBC patients with *EGFR* overexpression usually have worse overall survival and disease-free survival than normal expression patients. Therefore, *EGFR* overexpression is often associated with poor prognosis in TNBC [112,113]. Currently, the targeted therapeutic drugs that block the *EGFR* signaling pathway in breast cancer research are mainly anti-EGFR monoclonal antibodies (cetuximab). A phase II open randomized clinical trial evaluated the efficacy of platinum citrate cetuximab in patients with TNBC. The combination group had a 10% increase in complete release rate compared with the single-agent group, progression-free survival was extended by 2.2 months, and overall survival was extended by 3.5 months, but cetuximab may cause adverse reactions [95]. These cytological and clinical trials have demonstrated the enormous potential of EGFR inhibitors in the targeted therapy of TNBC.

### 5.6. Targeting Based on Cell Proliferation and Survival-Dependent Pathways in TNBC

#### 5.6.1. Targeting of the PI3K/AKT/mTOR Signaling Pathway

Distortion of the *PI3K/AKT/mTOR* signaling pathway is common in TNBC and has become a potential pathway for TNBC resistance to chemotherapy; phosphorylation of mTOR often suggests a poor prognosis in early TNBC [114,115]. Everolimus is an oral mTOR inhibitor that inhibits the downstream signaling of mTOR molecules in cells and arrests the cell cycle in the G1 or S phase, thereby inhibiting *PI3K/AKT/mTOR* pathway activity. A phase II clinical trial was performed in patients with TNBC (*n* = 50) treated with paclitaxel fluorouracil + epirubicin + cyclophosphamide (T-FEC), followed by randomization into the everolimus group (*n* = 23) and blank control group (*n* = 27). The results showed that the recurrence rate in the two groups was 47.8% and 29.6%, respectively, and the pCR rate was 30.4% and 25.9%, respectively [96]. Therefore, the study concluded that the pCR rate in patients with TNBC after combined treatment with everolimus did not improve significantly. In addition to monotherapy studies, a phase II neoadjuvant clinical study published in 2017 randomized TNBC patients into two groups; the study group (*n* = 96) was administered everolimus combined with cisplatin + paclitaxel, while the control group (*n* = 49) was administered placebo treatment. The results showed that the combination of everolimus with TNBC neoadjuvant therapy did not improve pCR or clinical response rates, but increased the incidence of adverse events [116]. Thus, inhibition of RTK upstream of *mTOR* leads to rebound activation of *AKT* [117]; conversely, inhibition of *AKT* activity initiates *FOXO*-dependent transcription and RTK activation [118]. Although blocking PI3K activity reduces *AKT* activation, it also leads to enhanced *MAPK* signaling [119]. In conclusion, this evidence is a good theoretical basis for studying dual inhibitors of the *PI3K/AKT/mTOR* pathway, which can control the activation of the target pathway and respond to its feedback loop, thereby preventing or delaying resistance. For example, combined mTOR and AKT inhibitors have shown synergistic efficacy in allograft models of basal-like patient origin [120]. A number of PI3K/mTOR inhibitors continue to be developed clinically, including gedatolisib (PF-05212384), which has therapeutic activity in breast cancer and acceptable safety and tumorigenic activity [121].

#### 5.6.2. Targeting of MAPK Signaling Pathway

Excessive activation of *MAPK* is involved in abnormal proliferation and apoptosis of cancer cells, which in turn contributes to TNBC malignancy. Ras and Raf mutations are infrequent in TNBC; instead, activation of *MAPK* signaling pathways is often thought to be caused by multiple mechanisms of upstream receptor tyrosine kinase activation or by activation or mutation of upstream proteins, such as *PI3K/AKT/mTOR* and related proteins [122]. Previous studies have shown that flutamide and C1-1040 have synergistic effects in the trastuzumab model, causing a decrease in ERK phosphorylation levels during combination therapy with trastuzumab [123]. One study found that ERK inhibitor in combination with Forskoli increased the sensitivity of TNBC cells to adriamycin [124]. In addition, PAD1 is used to treat metastatic breast cancer by regulating *MEK1–ERKl/2–MMP2* signaling in TNBC [125]. A growing body of preclinical evidence supports targeting the *Ras/MAPK* cell signaling pathway in TNBC subtypes, although large genomic surveys (TCGA public database) suggest that typical mutations in this pathway are rare [126,127]. Because of the early spread of TNBC, targeted treatment in the neoadjuvant setting may offer the effective therapeutic punch needed to eliminate micrometastatic disease and reduce mortality.

#### 5.6.3. Targeting the JAK2/STAT3 Signaling Pathway

In patients with TNBC, *JAK1*, and *JAK2* are usually overexpressed. The JAK/STAT pathway is involved in important biological processes such as cell proliferation, differentiation, apoptosis, and immune regulation. Phosphorylated *STAT3* is found in more than 50% of breast tumors and is associated with invasive phenotypes and poor prognosis [128]. Previous studies have found that abnormalities in the *IL-6/JAK2/STAT3* pathway play a crucial role in TNBC [129]. This suggests *JAK2/STAT3* to be a potential therapeutic target. The JAK1/2 inhibitor ruxolitinib is now approved for the treatment of myelofibrosis and is expected to improve the treatment of TNBC as a single-targeted drug [130].

#### 5.6.4. Targeting of the Src Signaling Pathway

**Figure 2:**
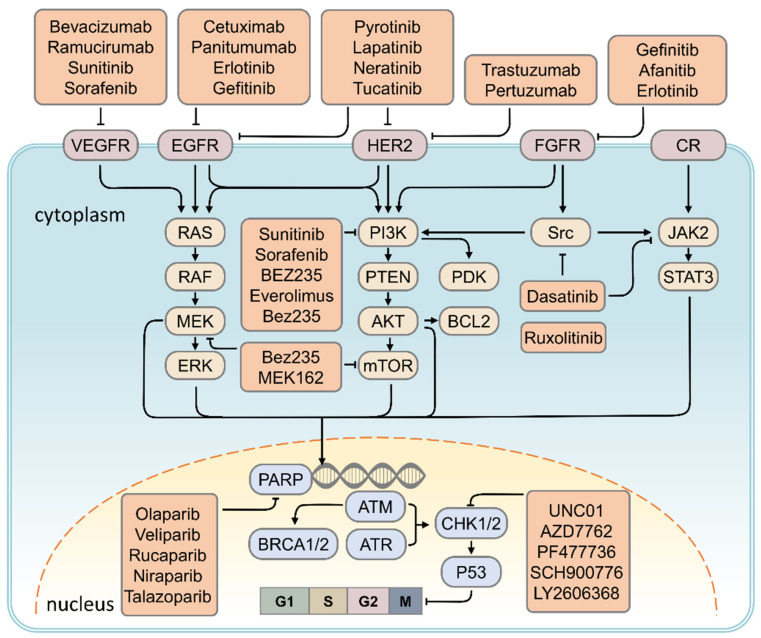
Schematic drawing presents the detail signaling pathways of breast carcinogenesis. All signaling pathways represented in this diagram have been found to be operative in the breast cancer cell context, whereas evidence to date supports a predominant role of HER2 in BRCA1/2 signaling and EGFR–PARP cross-talk in breast cancer cells. CR: cytokine receptor.

*Src* tyrosine kinase is overexpressed in TNBC and is involved in disease progression. Preclinical studies have shown that the combination of Dasatinib and cisplatin, a combination of *Src*, *c-ki*t, and *PDGFRβ*, can inhibit the growth of basal-like tumor cells or TNBC tumor cells [131,132,133]. In a phase II clinical trial using Dasatinib, anthracyclines, and paclitaxel in combination for treatment of metastatic TNBC, disease control rates were found to be 9.3%, partial response rates were 4.3%, and progression-free survival was around 8.3 weeks [100,101]. (Figure 2)

## 6. Immune Checkpoint Blockade

The tumor microenvironment is important for TNBC to achieve its biological behavior. It is not only the battlefield of immune surveillance and immune escape between tumor cells and the organism, but also the site where many prognosis-related effector cells, cytokines, and inflammatory mediators function, and the site of host immune killing and tumor immune tolerance [134,135]. Immune checkpoint inhibitors (ICBs) can inhibit the activity of immune checkpoint molecules to release the activity of T cells and restore the killing effect on tumor cells for antitumor purpose. Currently, there are two main types of ICBs: CTLA-4 receptor inhibitors and PD-1/PD-L1 inhibitors. Expression of PD-L1 on tumor cells and on immune cells surrounding the tumor indicates that an antitumor immune response has originally occurred [136]. High expression of immunosuppressive molecules such as PD-L1, CTLA4, TIM-3, and IDO by cells in the tumor cell microenvironment is one of the mechanisms of immune escape from tumor cells [137,138]. PD-L1 and CTLA-4 are immune checkpoints expressed on the surface of antigen-presenting cells in the initiation and effector phases of T-cell activation, respectively. Previous studies have shown that approximately 50% of PD-L1 transmembrane proteins in breast cancer with androgen receptor (AR)-negative prevalence and TNBC cells express PD-L1, whose receptor is PD-1, and also bind PD-L2 as other ligands, and express *CTLA-4*. Both may coexpress *PIK3A* and *PTEN*, which may allow dual blockade of these pathways for the chance of a more effective tumor response [137,138,139]. Showing that *PD-L1* upregulation is more common in basal breast cancer and is associated with a higher T-cell cytotoxic immune response. *PD-L1* upregulation is associated with better survival and chemotherapy response. Reactivation of inactive TIL by PD-L1 inhibitors suggests that PD-L1 upregulation may be a promising strategy for breast cancer [140].

## 7. Conclusions and Future Perspective

In this review, we describe some of the main strategies for addressing the challenges in TNBC treatment. TNBC is a high-risk type of breast cancer with a high invasiveness, malignancy, recurrence rate, and heterogeneity. TNBC has unique molecular biological characteristics, and multiple signaling pathways are involved in the development of the disease. Research on TNBC has been extensive, and both in vitro investigations and clinical trials have yielded good results. However, no targeted drugs have been used in clinical treatment. The use of clinical medications requires more innovation, such as selecting drugs based on medical, genetic, and clinical factors specific to the patient, and making clinical medications more targeted, safe, and effective. There are still many obstacles left to overcome in the area of TNBC-targeted therapy in order to achieve personalized drug treatments for patients with TNBC.

## Figures and Tables

**Table 1:** BI-RADS mammographic assessment categories.

Category	Management	Risk of Cancer
Category 0: Need additional evaluation	Re-evaluation	
Category 1: Negative finding	Routine screening	Essentially 0%
Category 2: Benign lesion	Routine screening	Essentially 0%
Category 3: Probably benign lesion	Short-interval follow up	0–2%
Category 4A: Low suspicion of malignancy	Tissue diagnosis	2–10%
Category 4B: Moderate suspicion of malignancy	Tissue diagnosis	10–50%
Category 4C: High suspicion of malignancy	Tissue diagnosis	50–95%
Category 5: Highly suggestive of malignancy	Tissue diagnosis	≥95%
Category 6: Proven malignancy	Surgical excision	-

**Table 2:** TNM system of breast cancer.

When T	When N	When M	Clinical Stage
Tis	N0	M0	0
T1	N0	M0	IA
T0	N1mi	M0	IB
T1	N1mi	M0	IB
T0	N1	M0	IIA
T1	N1	M0	IIA
T2	N0	M0	IIA
T2	N1	M0	IIB
T3	N0	M0	IIB
T0	N2	M0	IIIA
T1	N2	M0	IIIA
T2	N2	M0	IIIA
T3	N1	M0	IIIA
T3	N2	M0	IIIA
T4	N0	M0	IIIB
T4	N1	M0	IIIB
T4	N2	M0	IIIB
Any T	N3	M0	IIIC
Any T	Any N	M1	IV

N1mi: nodal micrometastases.

**Table 3:** Targets and targeted drugs for triple negative breast cancer.

Signaling Pathway	Target	Target Drugs	Ref.
DNA repair pathway	PARP	Olaparib, Veliparib, Rucaparib, Niraparib, Talazoparib	[93]
EGF signaling	EGFR	Cetuximab, Panitumumab, Erlotinib, Gefitinib	[94,95]
Angiogenesis pathway	VEGF	Bevacizumab, Ramucirumab, Sunitinib, Sorafenib	[80,95,96]
PI3K/AKT/mTOR signaling	PDGF, PI3K, mTOR	Sunitinib, Sorafenib, BEZ235, Everolimus, Bez235	[96,97]
MAPK signaling	ERK, mTOR	Bez235, MEK162	[98]
JAK/STAT signaling	JAK2	Ruxolitinib	[99]
Cell cycle pathway	CHK1	UNC01, AZD7762, PF477736, SCH900776, LY2606368	[92]
Src tyrosine kinase signaling	Src	Dasatinib	[100,101]
Human epidermal growth factor receptor 2	HER2	Lapatinib, Neratinib, Tucatinib, Poziotinib, Pyrotinib, Trastuzumab, Pertuzumab	[102,103,104,105,106]
Fibroblast growth factor receptor signaling	FGFR	Gefinitib, Afanitib, Erlotinib	[107,108,109]
